# Intelligent Deep Learning and Keypoint Tracking-Based Detection of Lameness in Dairy Cows

**DOI:** 10.3390/vetsci12030218

**Published:** 2025-03-02

**Authors:** Zongwei Jia, Yingjie Zhao, Xuanyu Mu, Dongjie Liu, Zhen Wang, Jiangtan Yao, Xuhui Yang

**Affiliations:** College of Information Science and Engineering, Shanxi Agricultural University, Jinzhong 030801, China

**Keywords:** dairy cows, machine learning, keypoints, feature extraction, lameness

## Abstract

With the ongoing development of computer vision technologies, the automation of lameness detection in dairy cows urgently requires improvement. To address the challenges of detection difficulties and technological limitations, this paper proposes an automated scoring method for cow lameness that integrates deep learning with keypoint tracking. First, the DeepLabCut tool is used to efficiently extract keypoint features during the walking process of dairy cows, which enables the automated monitoring and output of positional information. Then, the extracted positional data are combined with temporal data to construct a scoring model for cow lameness. The experimental results demonstrate that the proposed method tracks the keypoint of cow movement accurately in visible-light videos and satisfies the requirements for real-time detection. The model classifies the walking states of the cows into four levels, i.e., normal, mild, moderate, and severe lameness (corresponding to scores of 0, 1, 2, and 3, respectively). The detection results obtained in real-world real environments exhibit the high extraction accuracy of the keypoint positional information, with an average error of only 4.679 pixels and an overall accuracy of 90.21%. The detection accuracy for normal cows was 89.0%, with 85.3% for mild lameness, 92.6% for moderate lameness, and 100.0% for severe lameness. These results demonstrate that the application of keypoint detection technology for the automated scoring of lameness provides an effective solution for intelligent dairy management.

## Simple Summary

This study proposes a novel cow lameness scoring method based on keypoint localization and temporal information to address the challenges associated with computer vision-based lameness detection. Utilizing DeepLabCut, keypoint features of cow walking are automatically tracked, and a lameness assessment model is established. The proposed method effectively tracks keypoints in visible light videos, fulfilling the requirements for real-time detection. Cows are classified into four lameness levels (normal, mild, moderate, and severe), corresponding to scores of 0, 1, 2, and 3, respectively. The method achieves an accuracy of 90.21%, with a keypoint extraction error of 4.679 pixels, demonstrating its potential for automatic and accurate lameness detection.

## 1. Introduction

Cow lameness is a common and serious issue in modern dairy farming, and it causes significant economic losses. Lameness arises from various pathological factors, e.g., interdigital dermatitis, sole ulcers, and laminitis, which severely damage cows’ hooves, leading to pain and affecting their basic physiological activities like walking, feeding, and drinking. Cow lameness also reduces milk production and reproductive capacity, and it can increase farm diagnostic and treatment costs, reduce the useful lives of cows, and force premature culling. Thus, efficient and effective identification of cow lameness is of economic and practical significance. Cow lameness impairs normal walking and can affect feeding and drinking, which results in the weight loss and decline of the immune system, thereby affecting production performance [1]. In commercial farming, cow lameness impacts the farm’s economic benefits directly. Previous studies have demonstrated that the milk production of lame cows typically decreases by 15–20%, and the reproductive capacity of affected cows is also affected considerably, leading to failed breeding and reduced calf birth rates. Thus, early identification and diagnosis of cow lameness can effectively mitigate the economic losses caused by the disease.

With the ongoing development of farming technology, the application of machine vision and deep learning technologies in animal behavior recognition has become increasingly widespread. Considerable research has achieved a lot of results in livestock body parameters and daily behavior recognition; however, research on cow lameness identification is still in its preliminary stage and faces certain limitations [2]. Typically, conventional cow lameness detection methods rely on manual observation. Farmers determine lameness by observing cows’ gait and behavior. Although this method is direct and easy, several issues, e.g., high subjectivity and low efficiency in manual observation, make it difficult to realize efficient and large-scale detection. In large-scale farms, manual monitoring frequently fails to identify sick cows quickly, which results in delayed treatment. In research on machine vision technology, previous studies have proposed automatic detection methods based on video analysis and image processing techniques. For example, Song Huaibo et al. [3] (2018) employed the NBSM-LCCCT-DSKNN (normal background statistical model local circulation center compensation track-distilling data of KNN) model to detect cow lameness by extracting the contour of a cow’s head and neck, fitting a straight line, and using the slope to judge if the cow is lame. However, this method does not function accurately for cows with small movement amplitudes or other abnormal behaviors. In addition, Van Hertem et al. [4] (2018) evaluated the performance of an automatic motion monitoring system that uses three-dimensional vision technology to monitor cow barns and extracts six feature variables from recorded video data. Here, the curvature angle and dorsal postural measurement around the dorsal side of the hip joint were used for lameness classification. This method can identify cow lameness effectively under certain conditions; however, in high-density feeding environments, the interactions between cows may affect video processing, leading to inaccurate image segmentation and feature extraction, which affects the accuracy of lameness identification. Kang Xi et al. [5] (2019) proposed a cow lameness detection method using a spatiotemporal difference algorithm that extracts binary images of cow hooves, studies the spatiotemporal characteristics of the walking behavior, locates the position where the cow hooves touch the ground, analyzes the movement sequence of cow hooves during walking, and extracts and classifies data on the position of the hooves on the same side for trajectory extraction. However, the recognition effect of this method in complex environments needs to be improved because the binarization of cow hoof images can lead to information loss, which affects the detection accuracy. Kang Xi et al. [6] (2021) used the characteristics of arched backs during lameness to detect lameness by calculating the curvature of the arched backs, and they achieved a detection accuracy of 90.0% on thermal infrared datasets and 83.3% on visible light datasets. However, a cow’s shoulders and hips have smaller areas, resulting in fewer distinct graphical features, and the presence of railings can cause obstructions, which reduces detection accuracy. Although cow lameness is primarily caused by hoof lesions, the hooves are in constant contact with the ground, thereby making hoof extraction relatively blurred [7]. However, discomfort when walking caused by hoof lesions in cows can radiate to various parts of the body, causing nodding and arched backs.

With the development of deep learning technology, researchers are attempting to combine deep learning methods and machine vision technology to realize automatic cow lameness detection. Deep learning can extract features from images automatically using neural network models and perform multilevel classification and recognition; thus, deep learning methods have great potential in animal behavior recognition tasks [8,9]. Therefore, this paper proposes a cow lameness detection method that combines machine vision and deep learning technology. The proposed method employs cameras to monitor the cows’ walking process in real time and extracts the key body part features of cows, e.g., hooves, heads, and backs, using image processing algorithms. The movement information of these body parts can reflect whether cows exhibit the symptoms of lameness. Specifically, a convolutional neural network (CNN) is used to process the cow’s motion images and extract the feature information of the key body parts. Then, a long short-term memory network is employed to analyze the time-series data during walking to identify lameness behavior. Through this combined model, the real-time monitoring and automatic classification of cows can be achieved, categorizing cows into four levels: normal, mild lameness, moderate lameness, and severe lameness. The experimental results show that using this model can effectively improve the recognition accuracy of cow lameness and can be widely promoted in practical applications.

The automated detection of cow lameness is an important challenge in modern farming. By applying machine vision technology and deep learning methods, the efficient and accurate identification of cow lameness can be realized, which helps improve farming efficiency and reduce economic losses. Although current research has made progress, several challenges must be addressed, e.g., image processing issues in high-density feeding environments, the complexity of the cow’s body posture, and the differences in lameness manifestations caused by different etiologies. Thus, future research can further optimize algorithms, improve detection accuracy, expand its application to real-world production environments, and help the farming industry to better address the problems caused by cow lameness. Generally, cow lameness detection methods based on machine vision and deep learning have broad application prospects, and they are expected to provide important support for the management of modern farms and cow health monitoring.

## 2. Experimental Materials

### 2.1. Materials Source

**Figure 1:**
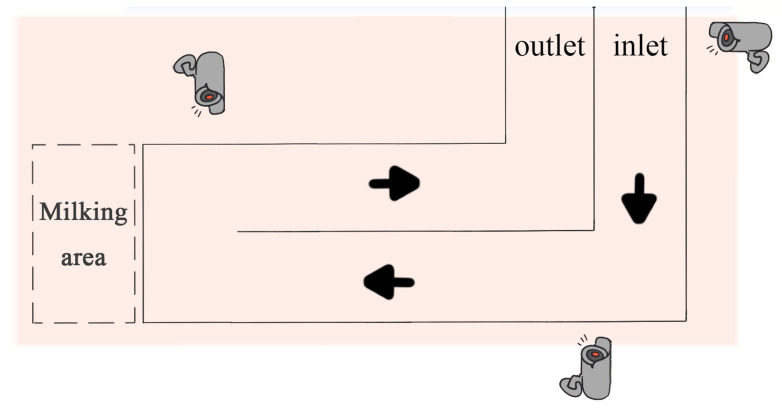
A schematic diagram of the limp video capture location.

The data collection environment of this study is illustrated in Figure 1. Dairy cows passed through a narrow passage before and after the milking process. The research team fixed the collection equipment at different locations on one side of the passage, where the distance between the camera (SONY: FDR-AX45, Static effective pixels: 8.29 million pixels (16:9)/6.62 million pixels (4:3)) lens and the passage was set at varying ranges from 1 to 5 m. Since the passage is not a straight passage, three cameras were used here to ensure that the cows were fully captured. Videos of the cows walking were captured from a side view to ensure that the captured video data were clear and complete. The data used in this study were collected in the morning, afternoon, and evening hours from May to July 2021, January 2022, and March 2023. Multiple video segments, ranging from 8 s to 2 min in length, were captured to obtain a rich sample of data. All videos were stored in AVI format with a frame rate of 25.00 frames per second (fps) and a resolution of 3840 × 2160 pixels. After screening and sorting, a total of 127 walking video segments, each approximately 10 s long, were obtained from 45 different cows (approximately 6 years old).

### 2.2. Data Preprocessing

**Figure 2:**
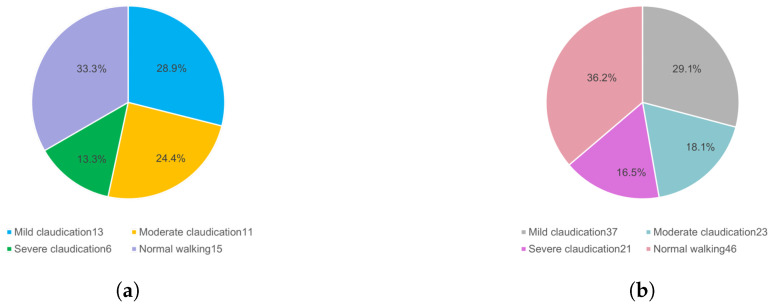
The number of cows with different degrees of lameness (**a**) and number of videos (**b**).

Three senior veterinarians classified the cows by observing the acquired videos. The number of cows with varying degrees of lameness in the data is shown in Figure 2a. As can be seen, normal and mildly lame cows accounted for more than 60% of the cows, and severely lame cows comprised approximately 10%. The types and quantities of the videos are shown in Figure 2b, indicating that the videos of normal walking and mild lameness were the majority, with nearly equal numbers of videos depicting moderate and severe lameness. In addition, accurate cow lameness detection was challenging due to the individual differences among the cows and the different movements during locomotion. To process the video data, the research team used Python tools (3.13.2) to capture one image in JPG format every 10 frames from each video, resulting in a total of 15,165 images. Then, to eliminate redundant data, the K-means algorithm was employed for image clustering to obtain images for labeling. To verify the effectiveness of this method, a comparative analysis was conducted using the uniform method. The results are shown in Table 1. The cow information appears in only the eleventh image with the uniform method, whereas it appears in the second image when using the K-means algorithm, significantly reducing the number of redundant images.

## 3. Keypoint Feature Extraction Method for Cow Walking

### 3.1. Keypoint Selection and Data Annotation

**Figure 3:**
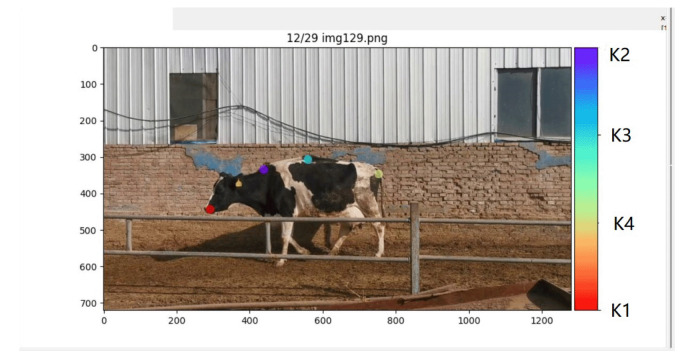
A diagram illustrating the positions of four key feature points.

Selecting appropriate walking key points is crucial in terms of the performance and accuracy of deep learning algorithms when applied to the cow lameness detection task. Reasonable keypoint selection provides precise information about posture and gait, including relevant information about the hooves, joints, and back. Monitoring the motion trajectories of these key points allows us to obtain detailed descriptions of the cows’ movements, which facilitates the development of accurate lameness detection models. Keypoint selection should be based on the anatomical structure and movement characteristics of the cow. Through an extensive literature review and traditional manual observation, this study identified typical postural features associated with lameness, including arched backs, head bobbing, leg swinging, slow speed, and asymmetric gait [10]. The research team marked one key point at the cow’s head and three key points along the back to construct a dataset for subsequent model training, as shown in Figure 3, labeled with labels K1, K2, K3, and K4. Here, K1 corresponds to the mouth area of the cow’s head, K2 corresponds to the back region near the neck, K3 corresponds to the mid-back, and K4 corresponds to the area near the tail. Ultimately, the research team selected 300 images from eight different cows as a labeled dataset, which was randomly divided into a training set (90%) and a testing set (10%).

### 3.2. DLC Backbone Feature Extraction Network Optimization

**Figure 4:**
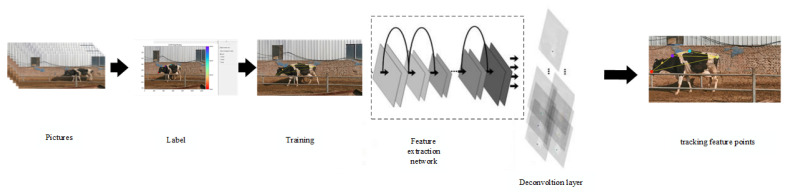
The workflow diagram of DeepLabCut.

This study utilized DeepLabCut (DLC), which is an open-source pose estimation and motion analysis tool [11] that accurately detects and tracks key points in images or videos. The DLC model comprises two key components, i.e., a pre-trained backbone feature extraction network and a deconvolution layer. The weights of the pre-trained feature extraction network are based on training from the ImageNet dataset, and the deconvolution layer samples visual information and generates the spatial probability density, which represents the likelihood of an object beinf at a specific location. To optimize the model for specific tasks, the research team fine-tuned the model weights on the labeled dataset comprising frame and body part position data with corresponding labels. During the training phase, the model weights were adjusted iteratively to enhance the prediction accuracy. As shown in Figure 4, the DLC model assigns high probabilities to marked body area locations and low probabilities to other locations [12,13,14].

**Figure 5:**
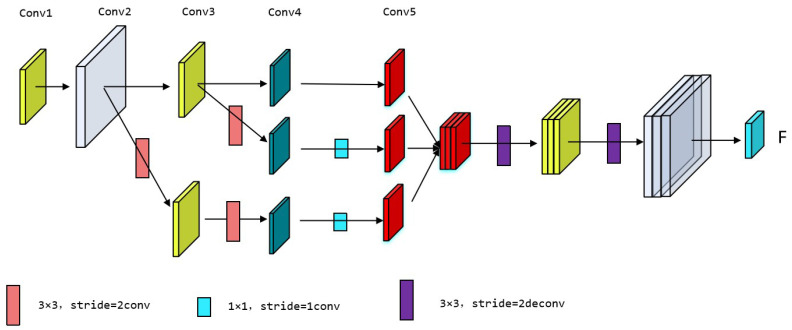
The structure of multiscale fusion.

To achieve an optimal detection model for cow back features, this study trained five pre-trained feature extraction networks: dlcrnet_ms5, ResNet-50 [15], ResNet-101 [16], MobileNetV2 [17], and EfficientNetV2 [18]. Note that these CNNs differ in structure and performance; however, they handle keypoint detection tasks effectively. ResNet-50 and ResNet-101 are classic CNNs that employ residual connections and deep residual structures; therefore, these networks can effectively address gradient vanishing and explosion issues while possessing strong feature representation capabilities. In contrast, ResNet-101 is deeper and performs better; however, it incurs higher computational costs. MobileNetV2 is a lightweight network that is suitable for mobile image classification and object detection, and it offers fast inference speeds. EfficientNetV2 optimizes performance and efficiency through adaptive network scaling across depth, width, and resolution. The dlcrnet_ms5 architecture integrates high-resolution features into low-resolution features to improve detection accuracy. As shown in Figure 5, this multiscale fusion module incorporates the high-resolution features from conv2 and conv3 into the low-resolution features of conv5. Before merging, these features undergo downsampling via a 1 × 1 convolution layer to reduce computational costs, which is followed by spatial upsampling through two stacked 3x3 deconvolution layers with a stride of 2. By integrating multilevel feature fusion and skeletal association techniques, the network can predict poses accurately, thereby enhancing the precision of pose estimation tasks.

### 3.3. Model Training and Evaluation

**Figure 6:**
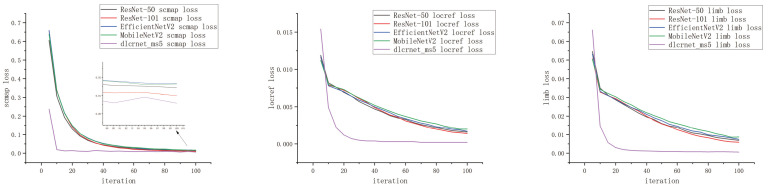
Loss curves.

During the training of the model, the Adam optimizer was selected with a batch size of one image. Here, the learning rate was initially set to 0.02 for the first 2000 iterations and then adjusted to 0.05. The entire training process consisted of 10,000 iterations, and the model’s metric parameter values were saved every 10 iterations. During training, the optimization of the model parameters involved three primary loss functions [19,20]. First, the location refinement loss (located loss), which is the core loss function in DLC, attempted to minimize the difference between the model’s predicted offsets and the true offsets to enhance the prediction accuracy of each body part’s location. Second, the score map loss (scrap loss) minimized the discrepancy between the model’s output probability map and the ground truth probability map, which ensured that the model could accurately locate each body part in the image. Third, limb loss was utilized to learn the connectivity between the body parts. During training, the model outputted the connectivity probabilities between each body part to represent their relationships in the image. The objective of the limb loss was to minimize the difference between the model’s output connectivity probabilities and the true connectivity probabilities, which enabled accurate predictions of the connections between the body parts. The variations in these three loss functions during the training process are shown in Figure 6. After training, the model’s performance was evaluated using the metrics described in Table 2 to ensure its effectiveness and reliability in key-point detection tasks.

Here, the “average Euclidean distance to GT per body part (in pixels)” (Table 2) refers to the global average distance in human pose estimation tasks. Initially, the Euclidean distance for each keypoint was calculated using Equation (1):

(1) $$d=\sqrt{{\left(x_{pred}-x_{gt}\right)}^{2}+{\left(y_{pred}-y_{gt}\right)}^{2}}$$

Here, ($x_{pred}$, $y_{pred}$) represents the keypoint coordinates predicted by the model, and ($x_{gt}$, $y_{gt}$) represents the true keypoint coordinates.

For each key point type (e.g., head, left hand, and right hand), the Euclidean distances of all key points of that type were summed and then divided by the total number of key points to calculate the average distance for that type. The global average distance was computed by summing the average distances of all keypoint types and dividing by the number of keypoint types, i.e., the average Euclidean distance to GT per body part (in pixels).

In addition, a comprehensive detection comparison was performed on an image sequence dataset of cows walking. Based on the results of the loss functions and the evaluation metrics, we found that although all five backbone networks had essentially converged after 10,000 training iterations, the dlcrnet_ms5 network exhibited the fastest convergence speed among the three loss functions and achieved the smallest final values. In terms of the performance parameters, the ResNet-101 network demonstrated the largest memory usage, and MobileNetV2 had the smallest. In terms of detection speed, the dlcrnet_ms5 model demonstrated superior performance with a detection speed twice that of the ResNet-50 network, which had similar memory usage. Although the five models exhibited comparable performance in terms of training loss for the Euclidean distance, the dlcrnet_ms5 network performed best in terms of test dataset loss. Thus, the dlcrnet_ms5 network was selected as the backbone network model for this study.

**Figure 7:**
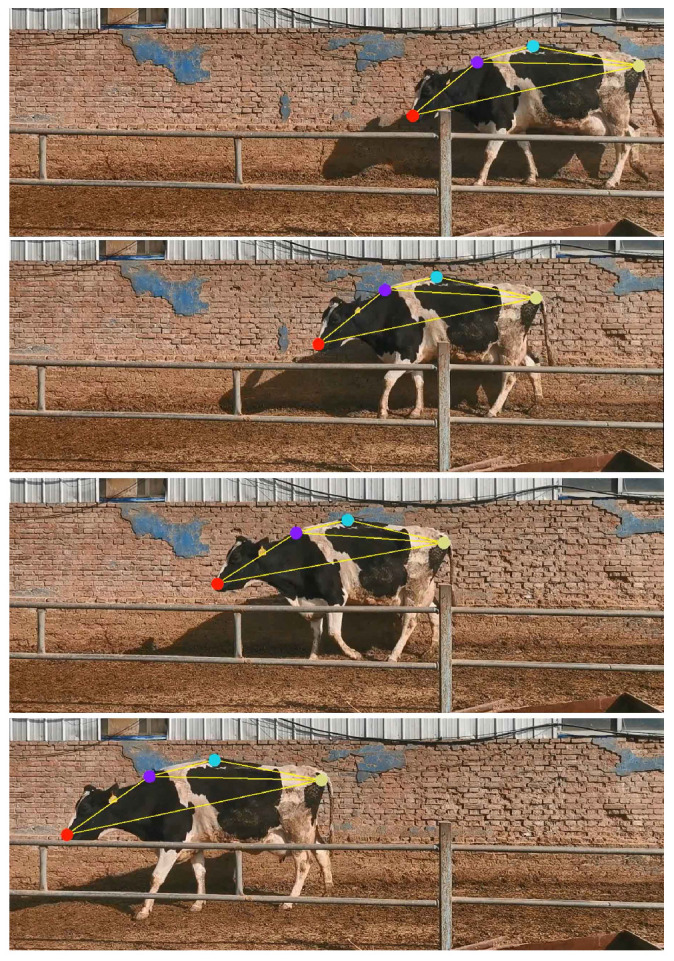
The tracking performances of the key points.

The keypoint tracking effect is shown in Figure 7.

## 4. Locomotion Scoring Methods

### 4.1. Analysis of the Trajectories of Key Points During Walking

This study quantitatively analyzed the position and movement of key points during the walking process of dairy cows. The trajectories of the key points for lame cows typically exhibit abnormal patterns and movement paths, which differ significantly from those of healthy cows [21,22,23,24]. Thus, the analysis of the trajectories of keypoints during walking is an important means to identify lameness in dairy cows. To this end, a walking video of a randomly selected dairy cow was used for the analysis of the keypoint trajectories and the trajectories of each keypoint during walking.

**Figure 8:**
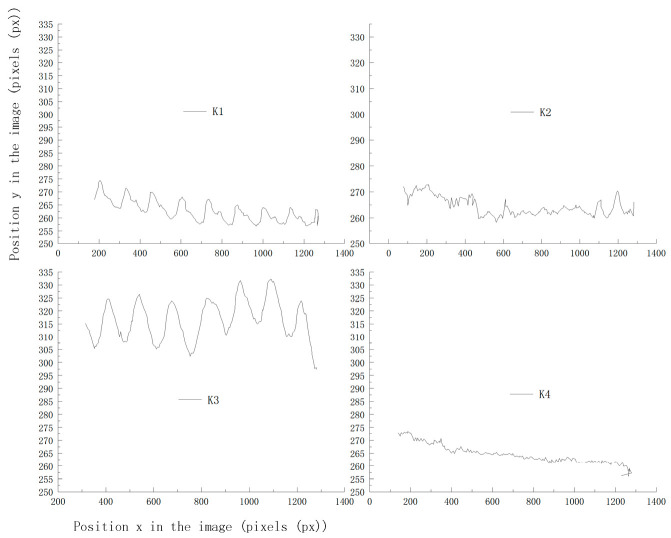
The trajectory plot of key points in space.

**Figure 9:**
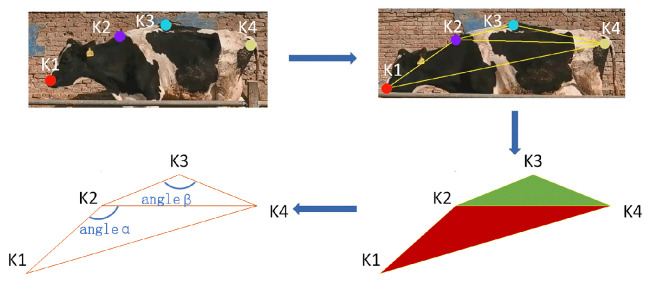
The characteristic triangle of the back and the characteristic angle.

Figure 8 shows the trajectories of the key points in two-dimensional space, where the vertical and horizontal axes represent the image’s vertical and horizontal dimensions, respectively, and K1, K2, K3, and K4 correspond to the different key points on the cow. It indicates that the trajectories of K1 and K3 exhibit regularity, and the trajectory of K2 exhibits significant fluctuations due to inaccurate recognition at the neck area. In contrast, K4 has a higher recognition accuracy. These results indicate that a single key point is insufficient for accurately judging whether a cow is lame. The gait of a cow involves a series of temporal signals, and these movements have a causal relationship in spacetime. Note that relying solely on positional information is inadequate to fully capture temporal characteristics. In addition, due to variations in individual body sizes and camera distances, the effectiveness of quantifying lameness based solely on key positions is limited. Based on these findings, this study captured the spatiotemporal movements of the feature points and performed an in-depth analysis of the gait characteristics. Here, feature triangles were constructed by integrating multiple factors, e.g., motion features and positional information, with K2 as the vertex, K1 and K4 as the endpoints of the base, and another triangle with K3 as the vertex, as well as K2 and K4 as the endpoints of its base (Figure 9).

During the construction of the characteristic triangle, the research team selected the coordinates of the cow’s lameness characteristic triangle and calculated the angular variations in two vertex angles α and β. The specific locations of angles α and β are shown in Figure 9. The location information of the key points is Ka ($X_{a}$, $Y_{a}$), and the distance La-b between every two key points is calculated using Equation (2). In addition, the angle α is calculated as shown in Equation (3), and the calculation method for angle β is the same.

(2) $$L_{1-2}=\sqrt{{\left(x_{1}-x_{2}\right)}^{2}+{\left(y_{1}-y_{2}\right)}^{2}}$$

(3) $$Angle\alpha =⊣\cos\left(\frac{L_{1-2}^{2}+L_{2-4}^{2}-L_{1-4}^{2}}{2L_{1-2}\;L_{2-4}}\right)\times \frac{180}{\pi }$$

**Figure 10:**
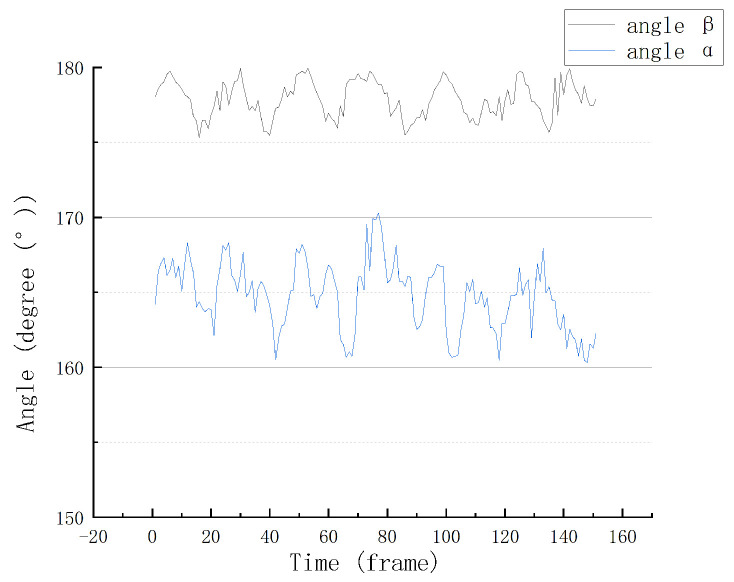
The feature angle change plot.

Taking the walking sequence of cow No. 005 as an example, the characteristic angular variations obtained after processing are shown in Figure 10.

As can be seen, the walking duration of the sampled cows was approximately 6s. In addition, the angle α of the sampled cows varied roughly between 160° and 170°, and the angle β varied between approximately 175° and 180°. The fluctuations in the changes in both angles were essentially consistent.

### 4.2. Development of Lameness Scoring Model

Establishing clear lameness scoring criteria is crucial to assess the degree of lameness in dairy cows accurately and implement effective intervention management. This standard can quantify lameness issues and provide objective evaluation indicators. Through a standardized scoring system, lameness issues can be classified into different severity levels ranging from mild to severe. This aids farmers in accurately assessing the degree of cow lameness and facilitates researchers in comparing and analyzing cows with varying degrees of lameness. The lameness scoring criteria provide a basis for guiding effective intervention management. Based on the scoring results, farmers can identify the severity and type of lameness issues and implement corresponding intervention measures. Upon reviewing the relevant literature, the identification of cow lameness typically refers to the classic three-point or five-point locomotion scoring system, with gait characteristics primarily including arched back, nodding head, leg flicking, slow pace, and asymmetric gait [25,26,27,28,29,30]. This study combined the three-point and five-point locomotion scoring systems to classify the walking status of dairy cows into four levels, as shown in Table 3.

**Figure 11:**
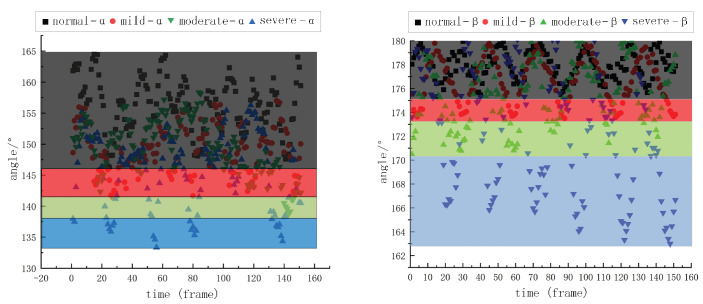
A diagram of angular α and β variation for different degrees of claudication.

Through the above process, we obtained the angular change value of the characteristic triangle of cows with different degrees of lameness, and we conducted an integrated analysis of the data from 45 cows. The results are shown in Table 4. The changes in angle α and angle β of cows with different lameness throughout 6 to 7 s (25 to 30 fps) are shown in Figure 11. It indicates that the more severe the lameness degree, the greater the angle variation, i.e., the more dispersed the value distribution. The ribbon colors in Figure 11 represent the range of the angular changes corresponding to each degree of lameness. Based on this finding, this study provides an effective evaluation method to assess the degree of lameness of dairy cows using the distribution of the feature angle values in a specific interval.

To establish the gait abnormality scoring model, refer to Equations (4)–(6). In the gait abnormality scoring model, scores are initially assigned based on the distribution of the single feature angle values within specific ranges, as shown in Table 5. Then, the initial scores of angles α and β are averaged to obtain the final score. In the case of decimal scores, the value is rounded up to the nearest whole number.

(4) $$S_{\alpha }=\left\{\begin{array}{cc} 0, & C_{\alpha }\in \left(0.75,1.00\right]\\ 1, & C_{\alpha }\in \left(0.50,0.75\right]\\ 2, & C_{\alpha }\in \left(0.25,0.50\right]\\ 3, & C_{\alpha }\in \left[0.00,0.25\right] \end{array}\right.$$

(5) $$S_{\beta }=\left\{\begin{array}{cc} 0, & C_{\beta }\in \left(0.90,1.00\right]\\ 1, & C_{\beta }\in \left(0.80,0.90\right]\\ 2, & C_{\beta }\in \left(0.70,0.80\right]\\ 3, & C_{\beta }\in \left[0.00,0.70\right] \end{array}\right.$$

(6) $$S=\left[\frac{S_{\alpha }+S_{\beta }}{2}\right]$$

In Equation (4), $C_{\alpha }$ denotes the degree of overlap between the variation interval of angle α for the tested cow and that for normal cows, and $S_{\alpha }$ denotes the initial score for angle α of the tested cow. In Equation (5), $C_{\beta }$ denotes the degree of overlap between the variation interval of angle β for the tested cow and that for normal cows, and $S_{\beta }$ denotes the initial score for angle β of the tested cow. In Equation (6), S denotes the final lameness score.

### 4.3. Model Testing

**Figure 12:**
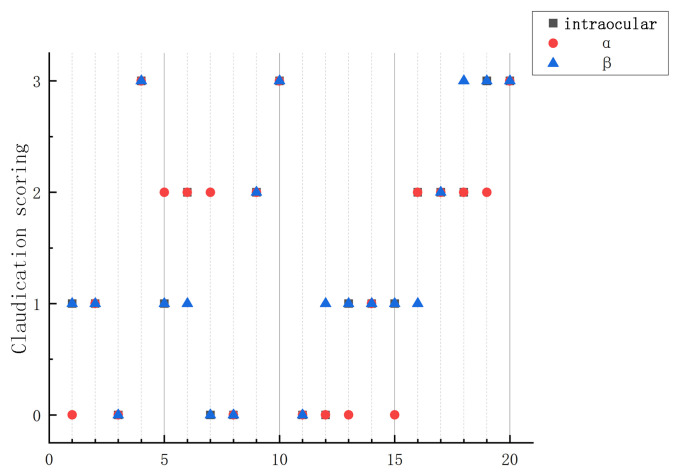
The model test scoring results.

In this study, 20 video files were randomly selected from 143 video files for testing, including 127 self-collected video files and 16 video files from other farms. The test results are shown in Figure 12. Among the test data, there is a significant difference in the results of Sample No. 7. After analyzing the corresponding video, we found that the cow was licking a railing while walking, thereby causing a large variation in angle α, which, in turn, affected the judgment of lameness.

After conducting gait scoring on all existing video data, the results indicate an overall accuracy rate of 90.21%. Specifically, the recognition accuracy for normal cows was 89.0%, that for cows with mild lameness was 85.3%, and that for cows with moderate lameness was 92.6%. In addition, the recognition accuracy for cows with severe lame-ness was 100.0%. Cows with moderate and severe lameness exhibited more pronounced back bending and nodding amplitudes, providing a higher degree of distinction. In contrast, the manifestations of normal cows and those with mild lameness were relatively similar, thereby resulting in a lower average classification accuracy compared with cows with moderate and severe lameness.

## 5. Conclusions

In this study, we investigated animal behavior recognition methods that primarily focus on animal outline contours and adopt key regional feature points as detection targets. This overcomes the difficulties of interference from various factors, e.g., cow body tremors and background changes, thereby reducing the number of feature points that need to be tracked and reducing the computational complexity of the algorithm.

The proposed method was evaluated experimentally, and the experimental results demonstrate that the method can accurately track cow walking keypoints in visible light videos and output positional information. Note that the proposed method is not affected by the distance between the cow and the camera lens and only requires the calculation of changes in the characteristic angles to assess the cow’s walking state. However, this study still has limitations in cow lameness detection and scoring; it is reflected in the test that the cows all return to the barn directly after coming out of the milking workshop, and the cows all walk at a normal speed without considering the slow step, fast “gallop” state. On the other hand, if several cows walk in a row, the system cannot handle them individually, so only one cow can be in the observation area at a time. To improve the accuracy of feature extraction, future studies should explore ways to introduce more walking characteristic data and utilize technologies, e.g., transfer learning. In addition, the generalizability of the model must be verified and enhanced for different breeds of cows and diverse farming environments.

## Figures and Tables

**Table 1:** The frame splitting method and effect demonstration.

Frame Splitting Methods	Effect									
Uniform	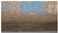	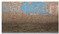	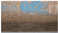	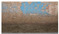	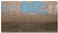	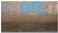	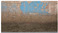	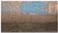	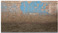	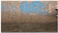
	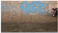	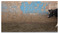	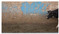	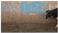	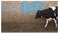	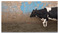	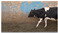	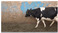	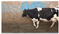	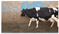
K-means	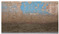	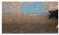	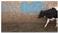	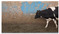	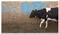	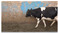	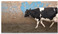	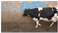	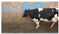	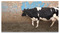
	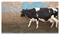	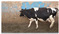	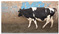	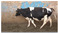	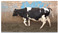	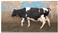	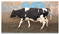	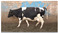	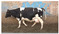	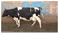

**Table 2:** The results of the model evaluation index.

Pretraining Model	The Memory Footprint of the Model/MB	Detection Speed/FPS	The Euclidean Distance Between the Predicted Key Point and the Real Labeled Key Point	
			Training Loss/Pixel	Test Loss/Pixel
ResNet-50	90.0	11.6	4.227055	13.425434
ResNet-101	170.1	9.7	4.141327	15.245344
MobileNetV2	9.0	14.2	4.225975	14.435343
EfficientNetV2	13.7	12.4	4.07183	14.075312
dlcrnet_ms5	90.9	24.1	4.080373	5.145343

**Table 3:** Cattle lameness grading scale.

Level	Walking Status	Physical Manifestations
1	normal	Cows stand or walk with a straight back and a normal gait
2	mild	Cows stand with a straight back and walk with a bowed back with a normal gait
3	moderate	Cows stand and walk with a distinctly arched back, a short stride gait, and a nod
4	severe	Cows have difficulty walking, are unwilling to bear weight, and have a nod

**Table 4:** A table of characteristic angles of cows with different degrees of lameness.

	Argument	Normal	Mild	Moderate Claudication	Severe Claudication
Angle α	Variation interval	146.26–164.87	141.44–156.99	138.75–158.33	133.34–156.27
	Mean value	156.2779	149.0112	149.3532	145.9024
	Average amplitude	18.61	15.55	19.58	22.93
Angle β	Variation interval	175.29–179.99	173.53–179.93	170.48–179.95	162.99–179.95
	Mean value	177.8921	176.5915	175.4388	172.2169
	Average amplitude	4.70	6.40	9.47	17.96

**Table 5:** Lameness scoring table for cows.

Mark	Walking State	The Distribution of Feature Angle Values in a Specific Interval	
		Angle α	Angle β
0	normal	[75%, 100%]	[90%, 100%]
1	mild	[50%, 75%]	[80%, 90%]
2	moderate	[25%, 50%]	[70%, 80%]
3	severe	[0, 25%]	[0, 70%]

## Data Availability

Data are included in the text.
